# Medical education for equity in health: a participatory action research involving persons living in poverty and healthcare professionals

**DOI:** 10.1186/s12909-016-0630-4

**Published:** 2016-04-12

**Authors:** Catherine Hudon, Christine Loignon, Cristina Grabovschi, Paula Bush, Mireille Lambert, Émilie Goulet, Sophie Boyer, Marianne De Laat, Nathalie Fournier

**Affiliations:** Department of Family Medicine and Emergency Medicine, Université de Sherbrooke, 3001 12e avenue Nord, Sherbrooke, QC Canada; Department of Family Medicine, McGill University, 5858 chemin de la Côte des Neiges, Montreal, QC Canada; Centre de santé et de services sociaux de Chicoutimi, 305 rue Saint-Vallier, Chicoutimi, QC Canada; Charles-LeMoyne Research Centre, 150 Place Charles-LeMoyne, Longueuil, QC Canada; ATD (All Together in Dignity) Fourth World Movement, an international community organization against poverty, 6747 rue Drolet, Montreal, QC Canada; Academic Primary Care Unit Charles-LeMoyne, 299 boulevard Sir-Wilfrid-Laurier, Saint-Lambert, QC Canada

**Keywords:** Medical education, Primary healthcare, Equity, Poverty, Participatory research

## Abstract

**Background:**

Improving the knowledge and competencies of healthcare professionals is crucial to better address the specific needs of persons living in poverty and avoid stigmatization. This study aimed to explore the needs and expectations of persons living in poverty and healthcare professionals in terms of medical training regarding poverty and its effects on health and healthcare.

**Methods:**

We conducted a participatory action research study using photovoice, a method using photography, together with merging of knowledge and practice, an approach promoting dialogue between different sources of knowledge. Nineteen healthcare professionals and persons from an international community organization against poverty participated in the study. The first phase included 60 meetings and group sessions to identify the perceived barriers between persons living in poverty and healthcare teams. In the second phase, sub-committees deployed action plans in academic teaching units to overcome barriers identified in the first phase. Data were analysed through thematic analysis, using NVivo, in collaboration with five non-academic co-researchers.

**Results:**

Four themes in regard to medical training were highlighted: improving medical students’ and residents’ knowledge on poverty and the living conditions of persons living in poverty; improving their understanding of the reality of those people; improving their relational skills pertaining to communication and interaction with persons living in poverty; improving their awareness and capacity for self-reflection. At the end of the second phase, actions were undertaken such as improving knowledge of the living conditions of persons living in poverty by posting social assistance rates, and tailoring interventions to patients’ reality by including sociodemographic information in electronic medical records. Our findings also led to a participatory research project aiming to improve the skills and competency of residents and health professionals in regard to the quality of healthcare provided to persons living in poverty.

**Conclusions:**

Medical training and residency programs should aim to improve students’ and residents’ relational skills, more specifically their communication skills, as well as their awareness and capacity for self-reflection, by helping them to identify and recognize their biases, and limitations.

## Background

A growing body of evidence suggests that persons in the poorest socioeconomic groups have the greatest burden of illness [1–6], but also, experience the greatest difficulty in obtaining quality care [7–11]. In a Canadian Medical Association report based on 29 interviews with physicians from across Canada [12], they noted a lack of knowledge and skills to work with socioeconomically disadvantaged patients. In another study by Bloch [13], healthcare professionals with expertise in caring for persons living in poverty actually recognized that the average family physician does not have a substantive understanding of the daily reality of these people. The participants (i.e. physicians, nurse practitioners, and nurses with at least 2 years of experience providing care to homeless persons) in a study conducted by McNeil et al. [14], reported limited training, and thus limited understanding, of social determinants of health. The same issue was raised by participants in a study investigating the use of health-related services by Canadians with a low income [15, 16]. The investigators recommended sensitivity training to increase medical personnel’s awareness and understanding of the unique challenges, experiences, and needs of persons living in poverty. Hence, healthcare professionals and investigators concluded that there is a need for medical school curricula to adequately prepare future healthcare professionals to deal with poverty and its impacts on health and healthcare. But the perspectives of disadvantaged patients are rarely taken into consideration [17].

The objectives of our participatory study were to collaboratively: 1) explore the needs and expectations of persons living in poverty and healthcare professionals in terms of medical training on poverty and its effects on health and healthcare. Specifically, we sought to identify potential improvements to make to medical school curricula in order to better prepare physicians to deal with poverty and its impacts on individuals and organizations; and 2) identify actions in daily practice to overcome these barriers.

## Methods

### Study design

We conducted a participatory action research project in which both academics and those for whom the research results are intended (i.e., persons living in poverty and healthcare teams) collaborated closely in the research. Participatory research is a methodological approach that is increasingly recognized as useful for the involvement of stakeholders in the research process [18]. Israel et al. [19] defined participatory research as an approach that recognizes the socially constructed nature of scientific knowledge. Participatory research is also different in its involvement of researchers outside of the academic setting. These non-academic researchers—members of the community or representatives of organizations—participate in all stages of the research. This equitable participation, based on a collaborative approach between the partners, allows the non-academic members to benefit immediately from the research findings or to become involved in knowledge transfer. In our study, the co-researchers were academic and non-academic members: two permanent volunteers of All Together in Dignity (ATD) Fourth World, an international community organization against poverty, four persons living in poverty, thirteen healthcare professionals, and eleven academic researchers.

This participatory study was part of a larger project that investigated barriers to quality and access to care for persons living in poverty by stimulating dialogue between persons living in poverty and healthcare professionals [20]. The EQUIhealThY project was developed as a result of our recent study indicating that physicians providing care in settings of poverty developed competencies that support effective care interactions with persons living in poverty [21]. A previous collaboration between CL and ATD Fourth World (MDL) facilitated the partnership for the EQUIhealThY project which was developed in collaboration with the two permanent volunteers of ATD Fourth World (MDL and SB) and the directors of the two participating academic primary care units (APCU). The aim of EQUIhealThY project was to combine the perspectives of persons living in poverty and of healthcare providers to explore barriers to responsive care for underserved persons with a view to developing equity-focused primary care. The objectives were a) to examine factors that would encourage the involvement of persons living in poverty in the process of developing social competence in healthcare organizations, and; b) to identify actions required to promote the adoption of professional practices oriented toward social competence in primary healthcare teams.

Based on these objectives, our study comprised two phases. The first phase included 60 group sessions (steering and monitoring committee meetings, and photovoice and merging into knowledge sessions) with academic and non-academic researchers. The sessions served to identify the perceived barriers between persons living in poverty and healthcare teams. All the meetings lasted from 2 to 4 h. The second phase comprised the creation of local action plan sub-committees that brought together healthcare professionals and members of ATD Fourth World co-researchers. They identified two to three actions to overcome these barriers in their academic primary care unit.

The methods used in this study were photovoice and the merging of knowledge and practice. Photovoice, often utilised in participatory research, uses photography as a means of generating knowledge on the lived experience of participants [22]. This method “enables participants to take photos to elicit emotions, feelings, and insights about topics that may be shrouded in silence” (p. 376) [23]. It has been used in several healthcare research projects, but not so much in primary care research with health professionals [23]. The merging of knowledge and practice is a method developed by ATD Fourth World. This approach promotes the sharing of different perspectives by creating the necessary conditions for the meeting and dialogue between three sources of knowledge: academic and theoretical knowledge (i.e., academic co-researchers), action and engagement knowledge (healthcare professionals co-researchers) and existential and experiential knowledge (persons living in poverty co-researchers) [24, 25]. To build a common and comprehensive understanding, two meetings were held. In the first meeting, participants formed peer groups to collectively identify and formulate the research question and to build on their expertise. The second meeting was a large group discussion where participants shared their peer group discussions and their individual reflections. A group facilitator ensured all participants can express themselves and be understood, especially the most vulnerable.

To promote the involvement of different groups of stakeholders as co-researchers, we created a steering committee, which brought together four members of ATD Fourth World (two persons living in poverty and two permanent volunteers), two academic researchers (one is also a healthcare professional), one healthcare professional, and two research assistants. They made decisions at each phase of the study (e.g., choice of research question, data collection and analysis methods, participants, etc.), played a role in data analysis (e.g., identification of key themes, descriptive analysis of merging of knowledge, etc.) and also participated in writing a report on the qualitative analysis of the data. In case of discrepancy, co-researchers in the steering committee tried to reach a consensus. Otherwise, they asked for counselling from someone outside of the committee and familiar with the project and participatory action research.

### Setting and participants

This study was carried out simultaneously in two APCU, both of which have a dual mission: a population-focused mission of responding to the needs of the population in its territory in accordance with their status as a family medicine group in Quebec, and an academic mission to train residents. The two APCU were selected because they serve very different populations in terms of care experiences and poverty. In addition, these APCU are affiliated with the academic institution of the PIs (CH and CL). One APCU is in an urban setting, and its clientele includes people receiving social security, people with substance addictions, and immigrants living in poverty. This APCU worked in close partnership with ATD Fourth World. The other APCU is in a semi-urban setting far from the city centre, and serves a varied clientele that includes workers living in poverty, people benefiting from social security, and first generation immigrants.

Three groups of non-academic co-researchers participated in the study. The first included seven healthcare professionals from the APCU in an urban setting: two nurses, two physicians, two residents and one social worker. The second was composed of six healthcare professionals from the APCU in a semi-urban setting: one nurse, one physician, two residents, one psychologist and one administrative agent. The third group included six members of ATD Fourth World: four persons living in poverty and two permanent volunteers. All members of these groups participated in photovoice training.

### Data collection and analysis

Each member of the three groups was invited to take photographs, over a period of 4 weeks, that spoke to the steering committee’s research question: ‘What are the barriers between persons living in poverty and healthcare professionals?’ Subsequently, the group members met in their respective peer groups, with a trained facilitator, and presented their photographs. During these 2 to 4 h meetings, they shared their thoughts about the photographs, and chose which photographs and reflections to share with the other groups. Next, the ATD Fourth World group met with each healthcare professionals group during a half-day merging of knowledge and practice meeting. Sharing perspectives helped participants better understand each other and build common reflections. Both merging of knowledge and practice meetings were audiotaped and transcribed verbatim.

Data analysis was carried out in three steps and co-researchers (persons living in poverty, permanent volunteers of ATD Fourth World, healthcare professionals and academic researchers) were involved at each step. First, two research assistants developed a coding grid according to their first reading of the verbatim. Co-researchers reviewed and adapted it to produce a final version. Second, the coding grid was used by research assistants to code the transcripts using NVivo 9. Co-researchers reviewed and analysed part of the coding. Finally, the research assistants wrote a summary of the analysed data highlighting the themes that were generated and identifying the differences and similarities in the discourse of the healthcare professionals and persons living in poverty groups. Co-researchers contributed to the interpretation of the data and based on their coding, they also wrote two sections in the report on the qualitative analysis of the project data. The reliability of the analysis was enhanced through data triangulation and peer review. We integrated different sources of data, including transcripts from photovoice, merging of knowledge and practice and steering committee data. Peer review was ensured through meetings with an expert researcher on participatory approach as well as a research facilitator and numerous discussions and reflection among all co-researchers.

### Ethical considerations

This study was approved by the research ethics boards of the Centre de santé et de services sociaux de Chicoutimi and the Academic Primary Care Unit Charles-Lemoyne, Quebec, Canada (2011-035). All the participants completed and signed an informed consent form.

## Results

### Phase 1: Barriers between persons living in poverty and healthcare professionals in terms of medical training

Our study pointed out that many barriers exist between healthcare teams and persons living in poverty. Four themes emerged about medical training: (1) improving medical students’ and residents’ knowledge on poverty and living conditions of persons living in poverty; (2) improving medical students’ and residents’ understanding of the reality of persons living in poverty; (3) improving medical students’ and residents’ relational skills pertaining to communication and interaction with persons living in poverty; (4) improving medical students’ and residents’ awareness and capacity for self-reflection.

#### Improving medical students’ and residents’ knowledge on poverty and living conditions of persons living in poverty

Persons living in poverty insisted that healthcare professionals ignore their realities, perhaps due to limitations with medical training. For instance, one person living in poverty said: *For me, on this matter of training, there is also (…) the matter of lack of knowledge of the reality of poverty* while another mentioned that: *Health professionals don’t know the reality of your day-to-day life.*

Persons living in poverty also noted that most physicians are unaware of social security coverage:

*The coverage of various social security programs should be displayed in each doctor’s office. They have to know that, for example, Meals-on-Wheels at $6.75 per meal is too expensive for someone who lives on $600 per month, and even for somebody who has $900. These barriers are almost invisible because people don’t see them. It’s the lack of knowledge.*

#### Improving medical students’ and residents’ understanding of the reality of persons living in poverty

Persons living in poverty insisted that being aware of their reality and understanding it are two completely different things. Thus, it is not because one has knowledge of the situation that one automatically understands persons living in poverty. When a health care professional understands the reality of persons living in poverty, he or she is able to demonstrate an awareness and sensitivity to their conditions of living, to be empathetic to what they are experiencing, and to offer flexible treatment options adapted to their reality. Persons living in poverty complained that lack of knowledge leads to healthcare professionals’ lack of understanding of their situation:

*I do not know how you are trained today, if you have the time and the means to understand, but we’ve realized that we are very often faced with professionals who don’t understand us.*

Persons living in poverty and healthcare professionals also recognized that they think differently because they have different backgrounds, they live in different social contexts, and do not face the same realities. Therefore, they operate from different logics which bring them to make different life choices. One resident noted she does not always understand persons living in poverty choices:

*How much is a pack of cigarettes? How come someone who is living in poverty doesn’t immediately say: ‘I’ll quit smoking; thus I will have $250 left a month or something, and I can do lots of things with that money. And besides, it harms my health.’ So, we don’t understand […] there are lifestyles we can’t understand because we don’t have the same background.*

Both groups insisted that healthcare professionals’ understanding of those differences is very important because the treatments they prescribe need to be adjusted to the realities of persons living in poverty. As one resident noted:

*Sometimes, as* healthcare professional*, maybe we don’t see enough of the reality of the person to tailor our interventions to this reality (…) It is us, sometimes, the barrier between what we ask and the misunderstanding of what we ask, of what it means in their everyday life.*

**Fig. 1:**
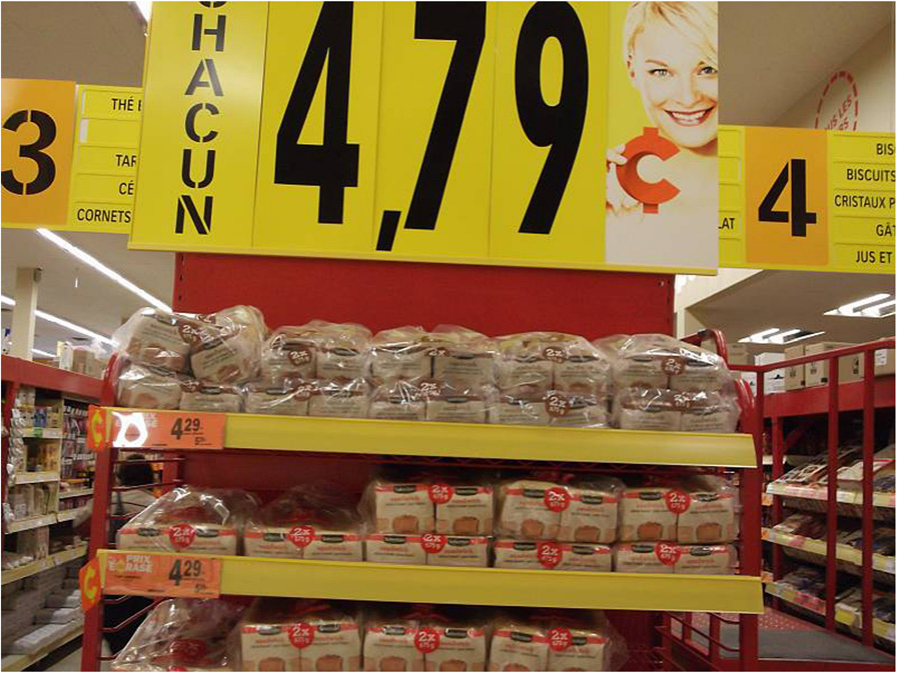
Bread loaves on a shelf. Photograph presented by a persons living in poverty to represent the barrier to a healthy diet and eating enough when you are living in poverty

A photo of bread loaves on a shelf with a banner showing the price (Fig. 1) referred to this incompatibility of living conditions of persons living in poverty with medical recommendations. Persons living in poverty know how to be healthy; they understand what healthcare professionals are trying to teach, but healthcare professionals should consider that persons living in poverty do not always have means and resources to be fit.

#### Improving medical students’ and residents’ relational skills pertaining to communication and interaction with persons living in poverty

Persons living in poverty emphasized the importance of physicians’ ability to establish relationships, and insisted that relational skills are just as important as technical skills: *We’ve talked a lot about medical training. We do not know how you’ve been trained, but we think that the communication aspect, the relationship, is as important as the scientific training.*

**Fig. 2:**
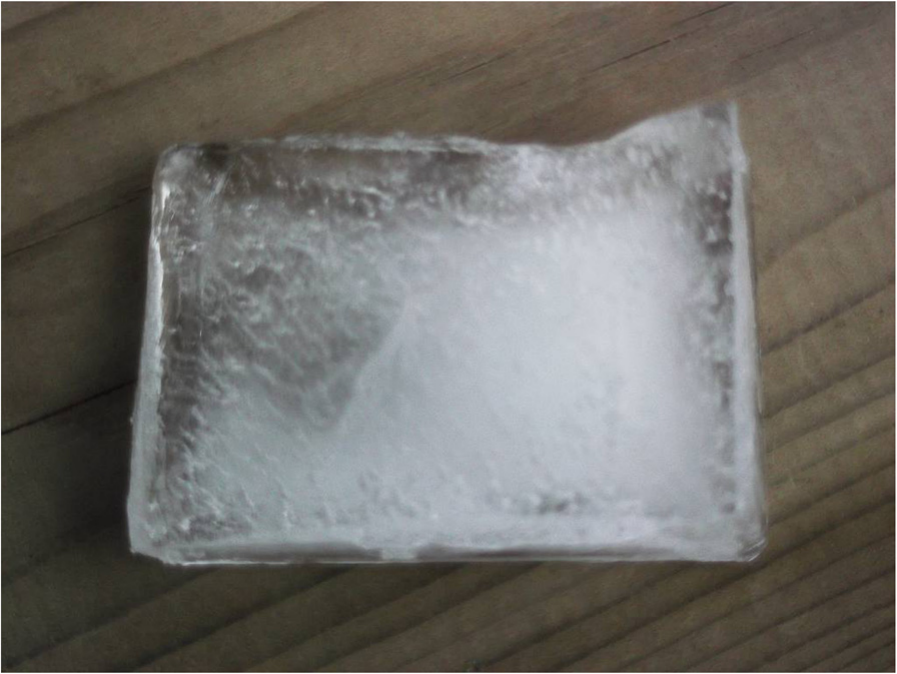
Block of ice. Photograph presented by a persons living in poverty to represent the limited relational skills of physicians

Another person living in poverty said, referring to the photo of a block of ice (Fig. 2):

*A block of ice. This is a physician who has received scientific training, who’s a good physician, but who’s unable to communicate. In medical training, it’s about choosing the right students. Communication is as important as the science. The ability to enter into a relationship with people—the barrier is the medical training.*

Healthcare professionals mentioned that over the last decade, there has been an increase in communication training and courses focusing on the importance of relationships with patients, but it is too early to see the effect of this training. Healthcare professionals also added that they often lack the time to build relationships with their patients, indicating they feel a tension between the need to make a proper diagnosis and the need to adequately communicate and engage with the patient. Indeed, one resident expressed that healthcare professionals often feel compelled to make a medical diagnosis without necessarily having the time and resources to take the overall situation of the patient into account:

*Yes, communication is important, the relationship with the patient and all that; but basically, I feel like we’re ‘diagnosticians’. (…) You have half an hour to analyze the patient and decide what you’ll do, because the next time you see him it may be in 6 months, or in 3 months. You know, it depends on our resources. It’s hard, (…) we can’t learn about the life of a person in half an hour, but we’re trying to do the best we can.*

In addition, healthcare professionals show some hesitation towards prioritizing the quality of the relationship at the expense of medical knowledge:

*Because they are bombarded with courses, they [the residents] have a lot of courses, and courses on physician-patient communication … never-ending courses; but all this considering that it’s a profession where you need to know many, many, many other things to be able to give you the correct diagnosis.*

#### Improving medical students’ and residents’ awareness and capacity for self-reflection

Persons living in poverty thought healthcare professionals are sometimes biased and may hold prejudices toward persons living in poverty, which can have a huge impact on their life: *[healthcare professionals] frequently make diagnosis based on prejudice without taking the time to consider the whole situation (…). Then, they trap us in this diagnosis, considered as a reference point for social workers and others healthcare professionals.*

Healthcare professionals’ biases needs to be recognized and eliminated, as one physician noted:

*We can’t forget, there are hundreds of residents who still have a ton of biases. And when I say ‘resident’ I think of all students who graduate in medicine, physiotherapy, occupational therapy, social work, I speak about them all; and I think: how can we reach these people and help them drop these biases? I think that over time, in practice, they end up eliminating them, but at what cost of personal suffering and suffering of others?*

Healthcare professionals recognized that they do not treat socioeconomically disadvantaged and more advantaged patients the same way. For instance, a resident said that she always insists that her patients living in poverty breastfeed their babies: *I think that breastfeeding is good for the poor as for the rich people, but I think I wouldn’t have the same discourse with both.* In addition, healthcare professionals may feel overwhelmed and powerless in the face of the complex problems of persons living in poverty, especially when they are young and inexperienced, as one physician admitted:

*When we arrive and we start as physicians it’s a big deal, we have a role (..) it’s a big hat, a cape, and we have to wear them (…) but somehow I think you must know that there is often great inexperience, sometimes a little immaturity (..) a lot of insecurity (…) but during training, we don’t always realize that (…) it’s not easy, we interact with people and sometimes there are people who have experienced situations so difficult that we are not necessarily super well equipped to deal with. So I think what is important that we improve our awareness, but it’s also important for you to know that we also experience a lot of insecurity, fear of failure,* etc. *(…) So you can’t forget that we have a lot of limitations too.*

A supervisor confirmed the importance of helping residents recognize their limitations:

*What we tell young physicians is, at least, know yourself. Know yourself and know your limits, and be honest with the patient. So, there are some who are able to do much more and go more into psychosocial aspects than others. But I think that for any patient, if he feels that the person is sincere: ‘listen … I'm overwhelmed by the situation, but I will try to find other resources’ (…) But, it takes a certain maturity and some self-criticism to make it there.*

### Phase 2: Actions to overcome barriers between persons living in poverty and healthcare professionals

At the end of the project, members of our team presented our findings to healthcare professionals, healthcare managers, APCU directors and medical education managers in each APCU and invited them to develop an action plan. This plan was submitted to persons living in poverty co-researchers who suggested some modifications. Each ACPU then adopted it. This plan included actions such as facilitating transport for persons living in poverty by distributing free public transit tickets, improving knowledge of the living conditions of persons living in poverty by posting social assistance rates, and tailoring interventions to patients’ realities by considering the patient’s socioeconomic conditions specified in their electronic medical records in the choice of their treatment intervention. Concerning this latter action, it is noteworthy that the International Organization of Medicine (IOM) has recommended the integration of social and behavioral data, such as financial resource strain, education and social isolation, into patient electronic health records [26]. This action could enable healthcare professionals to make more accurate diagnoses and engage more effectively with the patient in making treatment choices.

Many healthcare professionals recognized that their participation in this project enhanced their level of sensitivity concerning the conditions of persons living in poverty. The latter expressed that they were more aware of the medical practice context and care organizations barriers in the healthcare system. Interactions between APCU and community organizations were increased. Our findings also led to a participatory research project aiming to improve the skills and competency of residents and health professionals in regard to the quality of healthcare of persons living in poverty. A masters student in our team is currently working with persons living in poverty to involve them in the medical education of residents.

## Discussion

The objective of this research was to collaboratively explore the needs and expectations of persons living in poverty and healthcare professionals regarding medical training on poverty and its effects on health and healthcare, a subject that has been only sparingly documented in the literature. The results revealed that there is a need to improve medical students’ and residents’ knowledge and understanding of poverty and the living conditions of persons living in poverty. Medical training and residency programs should also aim to improve students’ and residents’ relational skills, specifically communication, and their awareness and capacity for self-reflection, by helping them to identify and recognize their biases and limitations.

Our findings on the fact that medical training is lacking in the area of teaching residents how to deal with the complex situations of persons living in poverty with confidence are corroborated by other studies suggesting that both healthcare professionals [12, 13] and persons living in poverty [15, 16] believe that more education about poverty and its effects is needed in medical training curricula. Medical schools should strengthen their curricula in such a way that future physicians are better prepared to deal with poverty and its impacts on health and healthcare. The question yet to be answered, however, is: what are the best methods to achieve these goals? Evidence shows that transient exposure to poverty (for instance, through lectures and one-time visits in clinics and organisations serving poor communities) is unlikely to adequately prepare medical students and residents to care for economically disadvantaged patients [17, 27, 28]. On the other hand, high exposure to poverty during medical training (e.g., studying at a teaching hospital located in a socioeconomically disadvantaged area) does not necessarily result in greater understanding and empathy either. In fact, it may lead to an erosion of students’ initially positive attitudes towards persons living in poverty throughout medical school [29–32].

The few medical and nursing schools that have successfully undertaken efforts to improve health professionals’ preparedness for caring for persons living in poverty, typically use an experiential learning approach where students are expected not only to provide direct service to persons living in poverty, but also to learn about, and reflect on, the context in which service is provided [14, 33]. The evaluation of such programs indicates that students who completed national and international clinical electives in settings providing care to persons living in poverty had better skills, knowledge and attitudes towards persons living in poverty populations, and also an increased desire to practice in underserved communities compared to students who had not completed such programs [34–43]. Notably, these studies report selection biases, indicating that typically it is the students with pre-existing interest and knowledge in the area who participate in these electives [27]. A potential solution is the implementation of mandatory elements in the curriculum that would provide medical students and residents opportunities to better understand poverty and the living conditions of persons living in poverty. Some North American medical schools have done this [27, 28, 44, 45] including curricular elements such as enhancement of course content [44], the introduction of multiple learning experience activities [27], and long-term involvement in projects developed with community-based organizations that serve disadvantaged populations [28, 45].

Despite the evidence that partnerships between medical schools and communities provide future physicians with the knowledge and skills to better serve underserved communities [33, 46, 47], we know of only one study that used the principles and practices of community-based participatory research to develop a medical curriculum that addresses responsive care related to poverty; it was conducted for internal medicine residents at Oregon Health and Science University [28]. In this study, the staff of a community-based organisation serving a homeless clientele was directly involved in the design and implementation of the medical curriculum, providing valuable expertise that faculty could not offer. Disadvantaged patients were also involved, but this was not explicitly described. Our study offers empirical evidence that supports the partnership model for which others have advocated [33, 46, 47] and that supplements the work of Gregg et al. [28]. Our results suggest healthcare professionals and persons living in poverty do not always have the same priorities, hence the importance of including vulnerable patients’ perspectives in the design of medical curricula.

Our study has some limitations. Our research comprised limited numbers of participants, but the sample size was appropriate given our participatory methodology [48] and allowed the study to benefit from the experience of non-academic researchers. Moreover our data reflect the experience of healthcare professionals and persons living in poverty in one area of one Canadian province (Quebec) with potential cultural differences compared to other countries. The results presented here may, thus, not be entirely transferable to all contexts. We also recognized that individual practitioners’ knowledge, understanding, and skills is only a small part of what is missing from a model of care that can appropriately respond to the needs of people living in poverty. Although we acknowledge this study, alone, does not fully reflect this complex reality, future research could explore driving forces, such as organisational, structural, and systemic factors, underlying accessible and quality care for persons living in poverty.

## Conclusions

There is a need to improve the knowledge and understanding of medical students and residents in regard to poverty and the living conditions of persons living in poverty. Medical training should also aim to enhance residents’ communication skills, awareness and capacity for self-reflection. Using the principles and practices of community-based participatory research could help to develop a medical curriculum that addresses responsive care related to poverty. Change in medical training represents a first step to improve health and healthcare of persons living in poverty, but further studies need to be developed to fully understand inequity in health. Participatory action studies with policy-makers and legislators could have great influence over the healthcare and benefits provided to persons living in poverty.

### Ethics approval and consent to participate

This study was approved by the research ethics boards of the Centre de santé et de services sociaux de Chicoutimi and the Academic Primary Care Unit Charles-Lemoyne, Quebec, Canada (2011-035). All the participants completed and signed an informed consent form.

### Availability of data and materials

Data is available on request from the corresponding author.

## Abbreviations

APCU: Academic primary care unit
ATD: All Together in Dignity
